# Translation, cross-cultural adaptation, and validation of the Integrated Palliative Care Outcome Scale Renal (IPOS-Renal) for Brazilian Portuguese

**DOI:** 10.1590/2175-8239-JBN-2025-0133en

**Published:** 2026-01-16

**Authors:** Márcia Alvarenga da Trindade, Ana Elizabeth Prado Lima Figueiredo, Carlos Eduardo Poli-de-Figueiredo

**Affiliations:** 1 Pontifícia Universidade Católica do Rio Grande do Sul, Medicina e Ciências da Saúde, Porto Alegre, RS, Brazil.; 2 Pontifícia Universidade Católica do Rio Grande do Sul, Enfermagem, Porto Alegre, RS, Brazil.; 3 Pontifícia Universidade Católica do Rio Grande do Sul, Medicina, Porto Alegre, RS, Brazil.

**Keywords:** Chronic Kidney Disease, End- Stage Kidney Disease Assessment Scales, Palliative Care, Nursing

## Abstract

**Introduction:**

Chronic Kidney Disease (CKD) is associated with a high symptom burden. In Brazil, there is no specific instrument to assess symptoms in CKD patients receiving palliative care. The Integrated Palliative Care Outcome Scale Renal (IPOS-Renal) has been translated and validated in 13 languages.

**Objective:**

To translate, cross-culturally adapt, and validate the IPOS-renal for Brazilian Portuguese.

**Methods:**

A methodological study conducted in eight phases: conceptual definition, forward translation, back-translation, expert review, cognitive interviews, proofreading, psychometric testing, and final reporting. A total of 153 stage 5 CKD patients were assessed with the IPOS-renal and the Edmonton Symptom Assessment System (ESAS-r) between February and March 2024. Instruments were reapplied after 15 days in 95 patients.

**Results:**

The scale demonstrated internal consistency, reliability, temporal stability, and convergent validity with the ESAS-r. The most prevalent symptoms were drowsiness (44%), difficulty walking (44%), fatigue/lack of energy (43%), and pain (36%). Less frequent symptoms included dyspnea (7%), diarrhea (14%), and vomiting (14%). Unbearable symptoms intensity was rare, including emotional symptoms (5%), difficulty sleeping (3%), and fatigue/lack of energy (3%). Symptom intensity varied, being severe for pain (24–25%) and drowsiness (21%); and moderate for difficulty walking (25%) and fatigue/lack of energy (22%).

**Conclusion:**

The Brazilian Portuguese version of the IPOS- renal is valid, reliable, and suitable for identifying biopsychosocial symptoms in stage 5 CKD patients receiving palliative care.

## Introduction

According to the World Health Organization^1^, kidney disease (KD) ranks ninth among the leading causes of global mortality. Between 2000 and 2022, the number of deaths related to chronic kidney disease (CKD) increased significantly, reaching 1.3 million people. Projections indicate that by 2040, CKD may become the fifth leading cause of death worldwide^2^.

The 2024 Brazilian Dialysis Survey^3^ revealed a 10% increase in the estimated number of patients undergoing dialysis compared to 2023, reaching 172,585 individuals and recording 29,712 deaths.

According to the Kidney Disease Improving Global Outcomes (KDIGO) guidelines, CKD is classified into six stages based on the estimated glomerular filtration rate (eGFR). In stages G1 to G4, conservative treatment is adopted, while in stage G5 — referred to as kidney failure or end-stage kidney disease (ESKD) — the initiation of renal replacement therapy (RRT) is indicated. These guidelines are essential for clinical management and the optimization of patient outcomes^4^.

Advances in nephrology have prolonged the survival of individuals with KD, contributing to an increase in the prevalence of CKD among the elderly. This population is characterized by multiple comorbidities and physical limitations that compromise their overall health status. Although RRT is essential for the management of advanced CKD, its benefits do not always result in improved quality of life. In addition to the limitations inherent to their renal condition, patients undergoing dialysis face challenges related to adapting to therapeutic demands, which often have a negative impact on their lives^5^.

Furthermore, in some cases, dialysis patients may express regret regarding the decision to initiate treatment. Therefore, it is crucial that the therapeutic plan focuses not only on extending survival but also on the patients’ well-being, incorporating shared decision-making based on clear information that addresses their values, expectations, and specific needs^6^.

In this context, palliative care is widely recommended, as its interdisciplinary approach considers the patient’s biography, values, and personal goals, placing them at the center of care^7^.

Palliative care aims to improve the quality of life of patients with chronic and life-threatening illnesses by alleviating suffering through the proper identification and management of symptoms. It also provides psychological, social, and spiritual support to both patients and their families. This approach strengthens the patient-caregiver bond, enabling early care planning and active participation in therapeutic decisions throughout treatment. Multidisciplinary teams play a key role in monitoring patients with advanced CKD, assisting in the development of comprehensive care strategies^8^.

The Palliative Care Quality Network analyzed a cohort of 33,183 hospitalized patients, all of whom underwent palliative care consultations during their hospital stay. Two groups were compared: patients with CKD, totaling 1,057 individuals (3.2%), and patients with other serious illnesses, corresponding to the remainder of the cohort. Both groups had similar clinical characteristics, including low performance scores (~35%), moderate to severe anxiety (~15%), and nausea (6%). After consultation, a general improvement in symptoms was observed, with a more pronounced reduction in anxiety in the group of patients with CKD (92% vs. 66%; p = 0.002). However, this group was less frequently referred to palliative care programs compared with patients with other serious diseases (30.7% vs. 37.6%; p < 0.001). Despite the high morbidity and mortality associated with advanced CKD, these individuals undergo palliative care less regularly, possibly due to a misconception regarding the need for such services^9^.

Many healthcare professionals have questions regarding the referral of patients to palliative care. To assist in determining eligibility, the University of Edinburgh developed the Supportive and Palliative Care Indicators Tool (SPICT), a measure designed to identify the need for palliative care in patients with advanced diseases. The SPICT-BR (Brazilian version) identifies people in the process of health deterioration, including specific indicators for CKD, such as stages G4 and G5, which are associated with clinical worsening. In addition, it considers KD as a complicating factor in other limiting conditions or treatments and addresses discussions about the decision not to perform dialysis in cases of clinical worsening or treatment intolerance. Thus, the SPICT-BR provides a more accurate and sensitive approach to the specific needs of patients with advanced CKD, allowing for timely interventions targeted at palliative care^10^.

There is a clear need for specific instruments to assess and plan care, enabling effective investigation of individuals’ needs. In the context of palliative care, the Edmonton Symptom Assessment System (ESAS-r)^11^ is widely used to measure symptom intensity. This scale classifies symptoms as mild (0 to 3), moderate (4 to 7), or severe (8 to 10), thereby assisting in the analysis of clinical complexity. In addition, it provides a solid foundation for the development of individualized intervention strategies^12^.

Loss of functionality is a striking feature among patients receiving palliative care^13^. In this context, assessing functional performance is essential for estimating patient prognosis. The Palliative Performance Scale (PPS) assesses mobility, activity and evidence of disease, self-care, food intake, and state of consciousness, assigning scores ranging from 0% (death) to 100% (full functionality). In addition to providing a clinical overview, this scale can be integrated into more comprehensive prognostic assessments^14^.

However, neither the PPS nor the ESAS-r are scales specifically designed to assess symptoms in patients with chronic kidney disease (CKD) receiving palliative care, and, to date, there is no instrument in Brazil specifically targeted to this population. In the international context, the Cicely Saunders Institute, King’s College London (KCL-CSI), developed the Integrated Palliative Care Outcome Scale Renal (IPOS-Renal), a disease-specific instrument for the comprehensive assessment of symptoms in patients with advanced CKD receiving palliative care. This scale has been translated and cross-culturally adapted into thirteen languages^15^.

Thus, this study aimed to translate, validate, and cross-culturally adapt the IPOS-Renal into Brazilian Portuguese.

## Methods

The study was designed as a methodological investigation aimed at translating, validating, and cross-culturally adapting the IPOS-Renal into Brazilian Portuguese.

The authors followed the guidelines established by KCL-CSI to ensure the effectiveness of the process. The study was conducted in eight phases, described below:

**Phase I – Conceptual definition:**

The conceptual definition was conducted in three stages:

I) Literature review on the quality of life of patients under palliative care.
II) A meeting involving seven palliative care specialists — two physicians, three psychologists, a nurse, and a spiritual counselor. The group evaluated and discussed each item of the IPOS-Renal, reaching a consensus on its suitability and relevance for clinical practice.
III) Using two groups of patients with advanced CKD, we investigated whether the version of IPOS-Renal defined in Phase II would be understood by patients. Three hemodialysis patients and two peritoneal dialysis patients were selected, and the IPOS-Renal was adjusted based on the participants’ suggestions.

**Phase II – Forward translation into Brazilian Portuguese (conducted by two translators and one mediator):**

At this stage, two native Brazilian translators independently translated the original English version of the IPOS-Renal into Portuguese. Subsequently, a mediator received both versions and synthesized them into a single document.

**Phase III (blinded) – Backward translation from Portuguese into English:**

Two native English-speaking translators performed the back-translation of the Portuguese document produced in Phase II, without having access to the original version. Following this stage, the mediator was again involved, receiving the two translations and synthesizing them into a single document.

**Phase IV – Review by experts:**

A group consisting of all those involved in the process was formed: healthcare professionals, translators, the mediator, and the researcher. After extensive discussions, consensus was reached on the pre-final version of the IPOS-Renal, which was then submitted to KCL-CSI for review. The KCL-CSI suggested some adjustments, which were discussed and agreed upon by the researchers. Subsequently, the document was submitted for reevaluation by the KCL-CSI, with the aim of obtaining approval of the pre-final version.

**Phase V – Cognitive interview – pre-test of the IPOS-Renal in Brazil:**

At this phase, the pre-test of the IPOS-Renal was conducted, involving ten patients at stage G5 and seven healthcare professionals. The objective was to assess the participants’ understanding regarding the items of the instrument. All individuals demonstrated full comprehension of each item, and no adjustments were deemed necessary for the final version.

**Phase VI – Proofreading:**

The final version of IPOS-Renal was submitted to the KCL-CSI for approval and authorization for psychometric testing to begin.

**Phase VII – Psychometric tests:**

For this study, the inclusion criteria were established as follows: adult patients aged 18 years or older, native Portuguese speakers residing in Brazil, and diagnosed with advanced CKD (stage G5) undergoing hemodialysis or peritoneal dialysis.

The exclusion criteria were: patients with communication difficulties, physical limitations that rendered them unable to participate in data collection, or an expected death within a few days. Cognitive assessment was performed using the Mini Mental State Examination, and those who exhibited cognitive impairment were excluded. In addition, patients who declined to participate in the study were respected^16^.

Patients were assessed using the newly adapted IPOS-Renal instrument, and the ESAS-r scale was employed as an external variable for convergent validity analysis. The correlation coefficient was applied, considering that both scales measure the impact and intensity of symptoms.

Data collection was conducted between February and March 2024, and included 153 patients with advanced CKD stage G5, 139 of whom were undergoing hemodialysis and 14, peritoneal dialysis. In 95 of these patients, the instruments were reapplied after a fifteen-day interval.

**Phase VIII – Reporting and publication:**

The final version of IPOS-Renal in Brazilian Portuguese has been officially published on the KCL-CSI website and is available for public access and clinical use.

### Ethical Aspects

The researchers adhered to the ethical and legal principles established by the Regulatory Standards and Guidelines for Research Involving Human Subjects^17^. The study was approved by the PUCRS Research Ethics Committee, under opinion No. 6,329,294. All participants signed the Free and Informed Consent Form, while the researchers signed the Data Use Agreement, undertaking to use the information exclusively for research purposes, maintaining the confidentiality and anonymity of all participants.

### Statistical Analysis

Data were organized in a Microsoft Excel® spreadsheet and analyzed using SPSS version 25.0, with a significance level of 5%. Descriptive statistics were applied (absolute and relative frequencies, means, medians, standard deviations, range, and interquartile range). The normality of the continuous variables was tested using the Kolmogorov-Smirnov method. The reliability of the instrument was assessed using Cronbach’s alpha coefficient (α), with values between 0.70 and 0.95 considered acceptable. The correlation between continuous variables was examined using Pearson’s or Spearman’s coefficients (in case of asymmetric distribution), classified as very weak (0–0.199), weak (0.200–0.399), moderate (0.400–0.699), severe (0.700–0.899), and very severe (0.900–1.0).

## Results

The results presented refer to a sample of 153 patients with advanced CKD stage G5. Patients were evaluated using a questionnaire comprising sociodemographic data and the application of the ESAS-r and IPOS-Renal scales. The results relating to the characterization of the sample are described in Table 1.

**Table 1:** Overall characterization of the sample (n = 153)

Variable	n	%	Mean	Deviation	Range
**Sex**					
Male	93	60.8			
Female	59	38.6			
Other	1	0.6			
Age (years)			57.8	13.3	24–82
Up to 29 years old	5	3.3			
From 30 to 39	12	7.8			
From 40 to 49	23	15.0			
From 50 to 59	36	23.5			
From 60 to 69	44	28.8			
70 or older	33	21.6			
**Marital status**					
Single	27	17.8			
Married	72	47.3			
Domestic partnership	21	13.2			
Divorced	17	11.2			
Widowed	16	10.5			
**Children**					
None	38	26.0			
1 to 2	69	45.0			
3 to 4	33	22.0			
Five or more	13	7.0			
**Education level**					
Illiterate	2	1.3			
Elementary education – complete	10	6.5			
Elementary education – incomplete	40	26.1			
High school – complete	39	25.5			
High school – incomplete	13	8.5			
Undergraduate degree – complete	26	17.0			
Undergraduate degree – incomplete	8	5.2			
Postgraduate degree – complete	9	5.9			
Postgraduate studies – in progress	1	0.7			
Master’s degree	3	2.0			
Doctoral degree (PhD)	2	1.3			
**Occupation**					
Specialists, intellectual/scientific occupations	38	24.8			
Technicians and associate professions	28	18.3			
Administrative functions and related positions	33	21.6			
Laborers, craft workers, and related occupations	28	18.3			
Domestic workers	12	7.8			
Others	14	9.2			
**Employment status**					
Employed	18	11.8			
Unemployed	2	1.3			
Retired	90	58.8			
On medical leave	29	19.0			
Self-employed	14	9.1			
**Place of residence**					
Inland areas of Rio Grande do Sul	7	4.6			
Coastal area	3	2.0			
Another state	1	0.7			
Porto Alegre	104	68.0			
Porto Alegre Metropolitan Area	38	24.7			
**Type of residence**					
House	107	69.9			
Apartment	45	29.4			
Long-term care facility	1	0.7			
**Number of cohabitants**					
None	22	14.4			
One	57	37.3			
Two	38	24.8			
Three	21	13.7			
Four or more	15	9.8			
**Religious affiliation**					
Yes	134	89.3			
No	19	10.7			
**Religious belief or affiliation**					
Believes in God/Jesus Christ	4	2.6			
Adventist	3	2.0			
Buddhist	1	0.7			
Catholic	77	50.3			
Catholic/Kardecist Spiritist	1	0.7			
Christian	7	4.6			
Kardecist Spiritist, *Candomblé, Nação*, and Catholic Church	1	0.7			
Spirituality, Kardecist Spiritist	15	9.8			
Evangelical	14	9.1			
Afro-Brazilian religion	12	7.7			
*Seicho-No-Ie*	2	1.3			
Jehovah’s Witness	3	2.0			
No religion and does not believe in anything	13	8.5			
**Time on therapy (years)**			3.5	3.6	15 days–22 years
Up to 6 months	28	18.3			
More than 6 months to 1 year	16	10.5			
More than 1 year to 2 years	29	19.0			
More than 2 years to 5 years	47	30.7			
More than 5 years	33	21.5			
**Type of vascular access**					
Graft	3	2.0			
Arteriovenous fistula	81	52.9			
Central venous catheter	55	35.9			
Peritoneal catheter	14	9.2			
**Comorbidities/Medical conditions**					
Systemic Arterial Hypertension	121	79.8			
Diabetes Mellitus	32	20.2			
**Number of other diseases reported**					
One	26	17.0			
Two to three	57	37.2			
Four to five	43	28.1			
Six or more	27	17.7			

Notes – A: Percentages obtained based on the total sample. Values presented as n (%) unless otherwise indicated. For continuous variables, the mean, standard deviation (SD), and range are reported. Percentages calculated based on the total sample (n = 153).

The sample consisted mainly of men (60.8%), with a mean age of 57.8 years (SD = 13.3), predominantly in the age groups of 60 to 69 years (28.8%) and 50 to 59 years (23.5%). Most participants were married (47.4%), had 1 to 2 children (42.1%), and had completed either elementary or high school education (both 34.0%). Intellectual/scientific occupations (24.8%) and administrative functions (21.6%) were the most frequent, with a predominance of retired individuals (58.8%). Regarding housing, 68.0% lived in Porto Alegre and 69.9% lived in houses; 37.3% lived alone and 24.8% with another person. Most reported having a religion (89.3%), predominantly Catholic (50.3%). The most common duration of hemodialysis or peritoneal dialysis was 2 to 5 years (30.7%), followed by >5 years (21.6%). The predominant vascular access was arteriovenous fistula (52.9%), followed by central venous catheter (35.9%). Among comorbidities, hypertension (87.6%) and diabetes mellitus (40.5%) stood out. The number of other diseases in addition to CKD varied, with 2 to 3 being the most frequent (35.6%), while 19.3% reported ≥ 6.

The IPOS-Renal results at the first assessment are depicted in Table 2 below:

**Table 2:** Absolute and relative distribution of ipos-renal items in the first assessment (n = 153)

Symptoms			IPOS-Renal Scale Classifications									
	Prevalence		No (0)		Mild (1)		Moderate (2)		Severe (3)		Unbearable (4)	
	n	%	n	%	n	%	n	%	n	%	n	%
**Symptoms reported on the scale**												
Pain	55	35.9	88	57.5	10	6.5	15	9.8	36	23.5	4	2.6
Shortness of breath	10	6.6	130	85.0	13	8.5	5	3.3	5	3.3		
Fatigue/lack of energy	65	42.5	55	35.9	33	21.6	34	22.2	26	17.0	5	3.3
Nausea/queasiness	34	22.2	102	66.7	17	11.1	15	9.8	15	9.8	4	2.6
Vomiting	22	14.3	123	80.4	8	5.2	8	5.2	10	6.5	4	2.6
Poor appetite	40	26.2	103	67.3	10	6.5	24	15.7	15	9.8	1	0.7
Constipation	27	17.6	117	76.5	9	5.9	12	7.8	11	7.2	4	2.6
Mouth sores/dry mouth	24	15.7	118	77.1	11	7.2	13	8.5	8	5.2	3	2.0
Drowsiness	67	43.8	63	41.2	23	15.0	32	20.9	32	20.9	3	2.0
Difficulty walking	67	43.8	68	45.6	18	11.8	38	24.8	24	15.7	5	3.3
Itching	33	21.5	94	61.3	26	17.0	19	12.4	12	7.8	2	1.3
Difficulty sleeping	49	32.1	81	52.9	23	15.0	24	15.7	20	13.1	5	3.3
Restless legs	25	16.4	106	69.3	22	14.4	15	9.8	9	5.9	1	0.7
Skin changes	28	18.3	105	68.6	20	13.1	15	9.8	10	6.5	3	2.0
Diarrhea	21	13.7	124	81.0	8	5.2	13	8.5	8	5.2	0	0.0
**Additional symptoms mentioned in open-ended questions**												
Pain/pain site	65	42.5	88	57.5	7	4.6	19	12.4	38	24.8	1	0.7
Emotional symptoms	25	16.3	128	83.7	1	0.7	1	0.7	16	10.5	7	4.6
Blood pressure- and blood volume-related symptoms	13	8.5	140	91.5	2	1.3	1	0.7	10	6.5	0	0.0
Gastrointestinal tract symptoms	16	10.5	137	89.5	3	2.0	1	0.7	9	5.9	3	2.0
During the last 7 days			No (0)		Rarely (1)		Sometimes (2)		Most of the time (3)		Always (4)	
Q3. Have you felt anxious or worried about your illness or treatment?	99	64.7	54	35.3			43	28.1	18	11.8	38	24.8
Q4. Have any of your family members or friends been anxious or worried about you?	134	87.6	13	8.5	6	3.9	28	18.3	29	19.0	77	50.3
Q5. Have you felt depressed?	44	28.8	100	65.4	9	5.9	27	17.6	3	2.0	14	9.2
During the last 7 days			Yes (0)		Most of the time (1)		Sometimes (2)		Rarely (3)		Never (4)	
Q6. Have you felt at peace?	46	30.0	87	56.9	20	13.1	17	11.1	2	1.3	27	17.6
Q7. Have you been able to share with your family or friends how you are feeling?	65	42.5	65	42.5	23	15.0	20	13.1	6	3.9	39	25.5
Q8. Have you received all the information you want?	22	14.4	122	79.7	9	5.9	15	9.8	4	2.6	3	2.0
During the last 7 days			Problems resolved or no problems experienced (0)		Problems mostly resolved (1)		Problems partially resolved (2)		Problems hardly resolved (3)		Problems not resolved (4)	
Q9. If you have experienced practical problems as a result of your illness, have they been resolved (such as personal and/or financial issues)?	63	41.2	71	46.4	19	12.4	52	34.0	4	2.6	7	4.6
During the last 7 days			None (0)		Up to half a day (1)		More than half a day (2)					
Q10. How much time do you estimate you spent on healthcare-related appointments (e.g., time spent traveling or repeating tests)?					65	42.5	88	57.5				
During the last 7 days			Alone (0)		With the help of a friend or family member (0)		With the help of a healthcare professional (153)					
Q11. How did you complete this questionnaire?								100%				

Notes – Prevalence defined as referring to symptoms classified as moderate, severe, and unbearable.

Following the administration of the IPOS-Renal scale, the most prevalent symptoms were drowsiness 43.8% (n = 67), difficulty walking 43.8% (n = 67), fatigue/lack of energy 42.5% (n = 65), and pain 35.9% (n = 55). The least frequent symptoms were shortness of breath 6.6% (n = 10), diarrhea 13.7% (n = 21), and vomiting 14.3% (n = 22).

At unbearable intensity, the highest percentages were reported for emotional symptoms 4.6% (n = 7), difficulty sleeping 3.3% (n = 5), and fatigue/lack of energy 3.3% (n = 5).

Among those classified as severe, pain/pain site (specific complaint regarding pain site) 24.8% (n = 38), pain 23.5% (n = 36), and drowsiness 20.9% (n = 32) predominated.

Among cases rated as moderate, the highest frequencies were difficulty walking 24.8% (n = 38), fatigue/lack of energy 22.2% (n = 34), and drowsiness 20.9% (n = 32).

The lack of symptoms was primarily observed for shortness of breath 85.0% (n = 130), vomiting 80.4% (n = 123), and diarrhea 81.0% (n = 124).

In the sections referring to the period “during the last 7 days”, approximately half of the sample, 50.3% (n = 77), reported that there is always a family member or friend who is anxious or concerned about the patient. Conversely, 65.4% (n = 100) stated that they did not experience symptoms of depression. Regarding a sense of peace, 56.9% (n = 87) stated that they felt at peace, and 79.7% (n = 122) reported having received all the information they wanted.

Regarding coping with practical problems resulting from the disease, the majority, 46.7% (n = 71), reported not having faced any problems to be solved, while 34.0% (n = 52) indicated that their problems had been partially resolved. As for the time spent on medical appointments, 57.5% (n = 88) of participants reported spending more than half a day.

In the statistical analysis, the new version of IPOS-Renal was tested for internal consistency, reproducibility (test-retest), construct validity, criterion validity (concurrent and/or predictive validity), and responsiveness to change. Cronbach’s alpha coefficient showed satisfactory internal consistency and reliability, demonstrating coherence among the items on the scale. The lowest internal consistencies demonstrated acceptable reliability, as shown in Table 3.

**Table 3:** Internal consistency analysis using cronbach’s alpha coefficient (if item excluded): ipos-renal (n = 153)

Symptoms	Scale mean if item is excluded	Scale variance if item is excluded	Corrected item-total correlation	Squared multiple correlation	Cronbach’s alpha if item is excluded
Q2_Pain	10.95	45.484	0.415	0.524	0.799
Q2_Shortness_of_breath	11.77	51.559	0.307	0.282	0.703
Q2_Fatigue_lack_of_energy	10.72	46.467	0.424	0.327	0.700
Q2_Nausea_queasiness	11.31	46.993	0.424	0.571	0.701
Q2_Vomiting	11.56	47.814	0.426	0.587	0.704
Q2_Poor_appetite	11.32	51.416	0.260	0.234	0.677
Q2_Constipation	11.48	54.580	0.360	0.275	0.698
Q2_Mouth_sores_or_dry_mouth	11.54	51.355	0.386	0.247	0.702
Q2_Difficulty_walking	10.80	46.211	0.420	0.340	0.722
Q2_Itching	11.31	51.783	0.139	0.227	0.678
Q2_Difficulty_sleeping	11.03	47.742	0.336	0.271	0.713
Q2_Restless_legs	11.48	50.225	0.290	0.283	0.721
Q2_Skin_changes	11.42	49.653	0.291	0.210	0.690
Q2_Diarrhea	11.64	52.258	0.161	0.211	0.692
Symptom1_Pain_Site	10.95	49.294	0.210	0.454	0.732
Symptom2_Emotional_Symptoms	11.50	51.712	0.204	0.166	0.645
Symptom3_Blood_pressure_and_blood_volume	11.80	52.978	0.127	0.230	0.737
Symptom4_Gastrointestinal	11.73	52.895	0.196	0.143	0.641

Notes – Cronbach’s alpha values ≥ 0.70 indicate satisfactory internal consistency; values between 0.60 and 0.69 may be considered acceptable in exploratory studies.

The mean time between test and retest was fifteen days. The evaluation revealed positive correlations, classified as weak (|0.200| to |0.399|) to moderate (|0.400| to |0.699|). The highest estimates were observed for the symptoms of poor appetite (r = 0.677), difficulty walking (r = 0.676), and difficulty sleeping (r = 0.681). Conversely, the lowest correlations occurred for emotional symptoms (r = 0.288) and shortness of breath (r = 0.328).

The ESAS-r is a questionnaire consisting of nine symptoms and an additional tenth option. Appetite and well-being scores were reversed. Mean symptom scores ranged from 0.60 (SD = 1.89) for nausea to 3.03 (SD = 2.41) for well-being, with most symptoms presenting as mild in intensity, with higher scores for drowsiness, appetite, and well-being. In the item for additional symptoms, complaints of pain/pain site, gastrointestinal symptoms, and emotional issues were reported.

The mean total score was 27.8 (SD = 10.2). However, the overall scale score in the test-retest showed no significant differences (p = 0.180), indicating stability between the mean scores of the initial (26.6 ± 9.8) and final (28.4 ± 11.1) assessments. The data for the ESAS-r scale are shown in Table 4.

**Table 4:** Mean, standard deviation, and median of the esas-r scale items in the initial assessment and retest (n = 95)

Items	ESAS-r Scale (n = 95)						
	Initial assessment			Final assessment			
	Mean	Standard deviation	Median	Mean	Standard deviation	Median	p^B^
ESAS_1Pain	0.91	2.15	0.00	1.60	2.66	0.00	0.234
ESAS_2Fatigue	1.36	2.96	0.00	2.20	3.12	0.00	0.014
ESAS_3Drowsiness	2.78	3.25	0.00	2.87	3.38	0.00	0.828
ESAS_4Nausea	0.55	1.73	0.00	0.32	1.44	0.00	0.310
ESAS_5WITH_Appetite_REVERSED	2.60	3.11	1.00	3.18	2.12	2.00	0.259
ESAS_6Shortness_of_breath	0.11	0.72	0.00	0.25	1.30	0.00	0.343
ESAS_7Depression	1.24	2.62	0.00	1.39	2.75	0.00	0.629
ESAS_8Anxiety	2.58	3.25	0.00	2.14	3.25	0.00	0.260
ESAS_9Well_being_REVERSED	2.93	3.03	2.00	2.64	2.68	2.00	0.068
ESAS_10Others^F^	1.98	2.01	2.00	2.06	1.96	2.00	0.075
ESAS_Total	26.6	9.8	24.0	28.4	11.1	28.0	0.180

Notes – B: Wilcoxon test. F: Others – Pain/pain site, emotional symptoms, and gastrointestinal tract symptoms.

For the analysis of convergent validity, ESAS-r was used as an external variable. The correlation coefficient was considered, since both scales assess the impact and intensity of symptoms. The results are presented in Table 5.

**Table 5:** Convergent validity between ipos-renal and esas-r using the spearman’s correlation coefficient

Symptoms	Spearman’s correlation coefficient (rS) – ESAS-r										
	Pain	Fatigue	Drowsiness	Nausea	WITH Appetite REVERSED	Shortness of breath	Depression	Anxiety	Well-being REVERSED	Others	Total
Q2_Pain	0.417**	0.098	-0.075	-0.014	-0.027	0.076	0.172*	0.194*	-0.068	0.220**	0.315**
Q2_Shortness_of_breath	0.114	0.237**	0.171*	0.056	-0.037	0.212**	0.050	0.177*	-0.108	0.205*	0.398*
Q2_Fatigue_lack_of_energy	0.222**	0.403**	-0.018	0.060	0.019	0.110	0.213**	0.243**	-0.141	0.305**	0.489**
Q2_Nausea_queasiness	0.008	0.177*	-0.009	0.185*	-0.197*	-0.068	-0.035	0.014	0.088	0.032	0.061
Q2_Vomiting	0.044	0.227**	-0.074	0.247**	-0.111	-0.090	0.050	0.165*	0.059	0.158	0.265*
Q2_Poor_appetite	0.030	0.071	0.100	0.067	0.009	0.207*	0.177*	0.012	-0.130	0.146	0.151
Q2_Constipation	-0.035	0.051	0.055	-0.004	0.041	0.172*	0.016	0.037	0.005	0.092	0.065
Q2_Mouth_sores_or_dry_mouth	-0.025	0.030	0.037	0.064	-0.105	0.074	0.104	0.173*	-0.007	0.149	0.111
Q2_Difficulty_walking	0.163*	0.108	0.003	0.168*	-0.002	0.166*	0.205*	0.215**	0.017	0.299**	0.502**
Q2_Itching	-0.037	-0.012	-0.123	-0.050	-0.097	-0.141	-0.048	-0.078	0.123	-0.032	-0.080
Q2_Difficulty_sleeping	0.043	0.068	-0.070	0.077	-0.033	0.096	0.116	0.189*	-0.046	0.152	0.137
Q2_Restless_legs	-0.050	0.090	-0.085	0.032	-0.052	-0.036	-0.076	0.042	-0.011	0.016	-0.010
Q2_Skin_changes	0.022	0.164*	-0.053	0.052	-0.050	0.160*	0.090	0.140	-0.107	0.148	0.120
Q2_Diarrhea	0.117	0.000	-0.027	-0.012	-0.025	0.124	0.112	0.199*	0.000	0.076	0.129
Symptom1_Pain_site	0.388**	0.049	-0.060	-0.003	0.049	0.059	0.132	0.111	-0.031	0.104	0.156
Symptom2_Emotional_Symptoms	0.071	0.018	-0.045	0.001	-0.073	0.238**	-0.003	0.060	0.196*	0.100	0.129
Symptom3_Blood_pressure_and_blood_volume	0.084	0.192*	-0.040	0.253**	0.054	-0.056	0.191*	0.104	-0.034	0.166*	0.389*
Symptom4_Gastrointestinal	0.083	0.032	-0.093	-0.050	0.063	0.169*	0.060	-0.108	0.016	0.035	0.007

Notes – *Significance at 5% (p < 0.05).

**Significance at 1% (p < 0.001).

For the convergent validity analysis of the IPOS-Renal, the ESAS was used as an external variable, considering the correlation coefficient between the scores of the two scales. As shown in Table 5, significant positive correlations were observed between the total ESAS-r score and IPOS-Renal symptoms, classified as moderate for fatigue/lack of energy (r = 0.489; p < 0.01) and difficulty walking (r = 0.502; p < 0.001). Significant weak yet relevant correlations were also found with pain (r = 0.315; p < 0.001), shortness of breath (r = 0.398; p < 0.01), blood pressure/blood volume-related symptoms (r = 0.389; p < 0.001), and vomiting (r = 0.265; p < 0.05). These results provide evidence of convergent validity for the IPOS-Renal in this sample.

## Discussion

CKD imposes a heavy burden of symptoms, which are often difficult to alleviate with conventional resources. Healthcare professionals may have difficulty recognizing certain symptoms, resulting in persistent untreated complaints. Thus, early identification and the implementation of appropriate strategies are essential to mitigate suffering^18^.

Despite the importance of routine symptom assessment, there are still gaps in clinical practice. Even when symptoms are reported, available treatments are limited and often not initiated, either due to the scarcity of specific evidence for this population or the complexity of drug interactions in kidney disease. Nevertheless, it is the responsibility of healthcare professionals to conduct a thorough assessment and provide effective individualized care^19^.

In the present study, the application of the IPOS-Renal revealed drowsiness, difficulty walking, fatigue, lack of energy, and pain as prevalent symptoms. These findings are consistent with the validation of the IPOS-Renal in Portugal^20^, which also identified pain (23.8%), poor appetite (23.1%), difficulty sleeping (17.9%), and fatigue/lack of energy (17.1%). In addition, they corroborate previous studies that highlight the frequency and impact of these symptoms in individuals with advanced CKD^21,22^.

In addition, patients’ perceptions regarding the time spent on consultations, tests, transportation, and commuting for treatment were investigated. Most participants reported spending more than half of their day on these activities, requiring rest upon returning home. Some mentioned the need to sleep, reinforcing the high prevalence of fatigue, tiredness/lack of energy, and drowsiness, as well as the impact of these symptoms on daily routines^23^.

A recent work conducted in Brazil supports the findings of this study. The research analyzed the impact of fatigue on the level of dependence among hemodialysis patients and found that 63 of the 101 participants (62.4%) experienced post-dialysis fatigue. This condition was associated with hemodialysis vintage (p = 0.041), recovery time after sessions (p < 0.001), and session shift (p < 0.001), being less frequent among individuals who underwent treatment in the evening^24^. These data converge with the reports of the interviewed patients, who mentioned using nighttime sleep as a way to recover from fatigue and lack of energy after nocturnal dialysis. In contrast, those who underwent treatment during the day reported needing to rest upon arriving home, which may have contributed to complaints of difficulty sleeping at night and daytime drowsiness.

In Denmark, the application of the IPOS-Renal revealed the prevalence of complaints regarding fatigue and reduced mobility, consistent with the findings of the present study. In addition, participants reported dizziness, memory changes, and sadness associated with dependence on family members and healthcare professionals—factors that contributed to social isolation and loss of identity^22,23,24,25^.

This study highlighted emotional symptoms, with 0.7% of complaints classified as mild, 0.7% as moderate, 10.5% as severe, and 4.6% as unbearable. The presence of emotional symptoms in dialysis patients has been confirmed by other authors, who emphasize the importance of early identification. Depression and anxiety, associated with family income, negatively impact the daily lives of these patients^26,27^.

In a study that analyzed the influence of psychopathological symptoms on the diagnosis, prognosis, and treatment response in renal failure, the application of the alexithymia scale revealed high scores, evidencing its association with these symptoms^28^. The term “alexithymia” was introduced in 1972 to describe patients with difficulties in expressing their emotions. It is composed of the Greek prefix *a*, which means “deprivation,” and *lexis*, which refers to *thymos*, or “mood”^29^. In the present study, patients demonstrated difficulty in naming their feelings regarding the changes caused by dialysis treatment. Although they denied complaints of depression (65.4%) and stated they felt at peace (56.9%), this denial contrasts with the distress expressed when they were questioned about their health conditions.

The first question of the IPOS-Renal questionnaire is open-ended, allowing patients to freely report their main problems or concerns over the past seven days. The questionnaire allows participants to indicate up to three responses. Based on the most frequent responses, three categories emerged: (1) concern about their own health in general, including symptoms and other comorbidities, in addition to CKD; (2) financial concerns; and (3) worries about family members and their health.

Regarding concerns about their own health, patients expressed anxiety, especially among those awaiting a transplant. This anxiety is manifested in the constant anticipation of receiving a phone call informing them of the availability of an organ, as well as in the uncertainty surrounding the clinical conditions required for inclusion on the waiting list. Furthermore, patients who had previously undergone transplantation but lost the graft and returned to dialysis experienced distress due to fears of ineffective treatment, particularly when witnessing the deaths of other patients in similar conditions during dialysis sessions.

These findings highlight the duality between the perception of well-being and underlying concerns, underscoring the need for an integrated approach that addresses both physical and emotional aspects in the management of advanced CKD. This perspective is essential for promoting a better quality of life for patients, considering the emotional complexities that permeate their experience throughout the course of treatment.

Patients with advanced CKD, although they have difficulty recognizing and clearly verbalizing their emotions, express their condition through feelings of stress, anxiety, worries, and fears, as well as symptoms such as difficulty sleeping, fatigue, and lack of energy. Data from this study corroborate the existing literature. One investigation identified depressive symptoms in 87% of patients, with 47% presenting severe or extremely severe levels of the disease and 66.7% in a severe or extremely severe state. Regarding stress, eight patients (53%) had no symptoms, while 27% were in a severe stage, and no patient had their stress classified as extremely severe^30^.

A study on the quality of life of patients with advanced CKD demonstrated that a symptom-oriented treatment plan significantly improves pain and depression. In addition, depression and fatigue affect self-management, reducing motivation for physical activity, adherence to dietary recommendations, and the ability to cope with a depressed mood^31^.

Another symptom identified in this study, after applying the IPOS-Renal, was the presence of pain in 35.9% of respondents, classified as mild in 4.6%, moderate in 12.4%, severe in 24.8%, and unbearable in 0.7%. Cicely Saunders, a pioneer in palliative care, coined the term “total pain” as she recognized, through patients’ narratives, that pain goes beyond the physical aspect, encompassing emotional, social, and spiritual dimensions^32^. Since then, palliative care practitioners have considered these factors in pain assessment, disseminating the concept of total pain and raising awareness among healthcare professionals about the need to understand pain as more than a subjective report, and to adopt effective measures of empathy and patient advocacy, ensuring adequate treatment, especially in healthcare settings^33,34^.

Uncontrolled symptoms negatively impact patients’ daily activities. A study involving 90 dialysis patients identified moderate-intensity musculoskeletal pain in 35.6% of respondents^35^. Furthermore, evidence indicates that pain compromises sleep quality^21,36,37^. These data are consistent with the findings of the present study and the extant literature, given that, among the additional symptoms of IPOS-Renal, patients reported complaints of pain in specific sites, such as the lower limbs, in addition to generalized body and joint pain.

To support the management of chronic pain, specialized palliative care teams can contribute with a comprehensive approach and assist in controlling persistent symptoms^8^. In the context of dialysis, pain control requires caution, especially in the choice of analgesics, due to the pharmacokinetic particularities of these patients^38^.

In a study conducted using the IPOS-Renal questionnaire, less prominent symptoms such as nausea and shortness of breath were identified. A similar finding was observed in the present study, in which the least frequent complaints were shortness of breath (7%), diarrhea (14%), and vomiting (14%)^25^.

However, the IPOS-Renal scale validated in Czech^19^ showed a prevalence of some symptoms that differed from those observed in this study. The most commonly reported symptoms included dry or sore mouth, nausea, poor appetite, and restless legs. Nevertheless, there were similarities in complaints regarding difficulty sleeping and weakness. This evidence reinforces the effectiveness of IPOS-Renal in identifying complaints in patients with advanced CKD.

To assess patient symptoms and test convergent validity, the ESAS-r instrument was used in addition to the IPOS-Renal. The results indicated the presence of symptoms such as anxiety, pain, altered well-being, reduced appetite, depression, drowsiness, and fatigue. It is noteworthy that symptoms of pain, drowsiness, and fatigue were also prevalent in the application of the IPOS-Renal, reinforcing the evidence of its convergent validity.

However, despite the available tools, challenges remain in identifying the needs of renal patients. In this study, it was observed that only one patient mentioned sexual dysfunction. Nonetheless, a research conducted in Brazil identified the prevalence of erectile dysfunction in 66.30% of individuals in a sample of 98 patients^39^.

Regarding women undergoing dialysis, a study involving 22 participants revealed that, although all were in stable relationships, only 7 (18%) maintained an active sex life, and no patients over the age of 60 reported sexual activity^40^. Therefore, it is essential to strengthen the relationship between patients and the healthcare team, providing a safe environment for them to express their needs.

Hemodialysis patients need to commute to treatment centers three times a week. This frequent contact with the professionals helps establish a bond with the multidisciplinary team, which may facilitate the addressing of more intimate complaints.

However, in this study, the researchers had no prior relationship with the interviewees, this being their first contact. This may have influenced the significant absence of reports on sexuality, constituting a limitation of the research.

In light of the above, the support of a multidisciplinary palliative care team is essential to assess and meet the needs of renal patients, especially as CKD progresses, assisting them in self-managing their disease and treatment.

In this context, IPOS-Renal, combined with the support of a team specializing in palliative care and the collaboration of the multidisciplinary dialysis staff, represents a valuable tool for identifying and implementing a therapeutic plan capable of mitigating patient suffering.

## Conclusion

The IPOS-Renal has proven to be a valid instrument, demonstrating good content validity, reliability, and sensitivity in detecting changes over time. Therefore, its application in Brazil is feasible for assessing the main symptoms of patients with advanced CKD undergoing palliative care. Its use not only enables a more accurate evaluation of patients’ needs, but also strengthens communication between the healthcare team and patients, promoting more person-centered care aligned with their values and preferences.

## Data Availability

The data supporting the findings of this study are available at reasonable request to the corresponding author.

## Supplementary Material

The following online material is available for this article:

Patient questionnaire – IPOS Renal.
