# Supplementary material for: Translation, cross-cultural adaptation, and validation of the Integrated Palliative Care Outcome Scale Renal (IPOS-Renal) for Brazilian Portuguese
Source: J Bras Nefrol. 2026 Jan 16;48(2):e20250133. doi: 10.1590/2175-8239-JBN-2025-0133en (PMC12810525; doi:10.1590/2175-8239-JBN-2025-0133en)
Supplement: Patient questionnaire – IPOS Renal. [file 2175-8239-jbn-48-2-e20250133-suppl1.pdf]

Brazilian Journal of Nephrology
https://doi.org/10.1590/2175-8239-JBN-2025-0133pt

Material Suplementar para "Tradução, adaptação transcultural e validação da escala *Integrated Palliative Care Outcome Scale Renal* (IPOS-Renal) para o português do Brasil"

IPOS- RENAL

QUESTIONÁRIO DO PACIENTE

Nome:

Por favor, responda às perguntas abaixo, escolhendo a opção que melhor representa como se sentiu nos últimos 7 dias. Sua contribuição é essencial para melhorar os cuidados oferecidos a você e aos demais pacientes com doença renal crônica. Obrigado (a)!

P1. Quais têm sido os seus principais problemas ou preocupações <u>nos últimos 7 (sete) dias?</u>

1.____

2.____

3.____

P2. Segue-se uma lista de sintomas que pode ou não ter tido. Para cada um destes sintomas faça um "X" na resposta que melhor descreve a forma como este sintoma <u>o/a afetou durante os últimos 7 (sete) dias?</u>

	Não	*Leve*	*Moderado*	*Forte*	*Insuportável*
**Dor**	0 ☐	1 ☐	2 ☐	3 ☐	4 ☐
**Falta de ar**	0 ☐	1 ☐	2 ☐	3 ☐	4 ☐
**Cansaço/falta de energia**	0 ☐	1 ☐	2 ☐	3 ☐	4 ☐
**Náuseas/enjôo**	0 ☐	1 ☐	2 ☐	3 ☐	4 ☐
**Vômitos**	0 ☐	1 ☐	2 ☐	3 ☐	4 ☐
**Pouco apetite**	0 ☐	1 ☐	2 ☐	3 ☐	4 ☐
**Intestino preso**	0 ☐	1 ☐	2 ☐	3 ☐	4 ☐
**Feridas na boca ou boca seca**	0 ☐	1 ☐	2 ☐	3 ☐	4 ☐
**Sonolência**	0 ☐	1 ☐	2 ☐	3 ☐	4 ☐
**Dificuldade para caminhar**	0 ☐	1 ☐	2 ☐	3 ☐	4 ☐
**Coceira**	0 ☐	1 ☐	2 ☐	3 ☐	4 ☐
**Dificuldade de dormir**	0 ☐	1 ☐	2 ☐	3 ☐	4 ☐
**Pernas inquietas ou dificuldades em manter as pernas quietas**	0 ☐	1 ☐	2 ☐	3 ☐	4 ☐
**Alterações na pele**	0 ☐	1 ☐	2 ☐	3 ☐	4 ☐
**Diarréia**	0 ☐	1 ☐	2 ☐	3 ☐	4 ☐
**Por favor, indique se houver quaisquer <u>outros</u> sintomas que não foram aqui referidos e faça um "X" na resposta que melhor descreve a forma como esses sintomas o/a afetaram <u>durante os últimos 7 (sete) dias.</u>**					
**1.**	0 ☐	1 ☐	2 ☐	3 ☐	4 ☐
**2.**	0 ☐	1 ☐	2 ☐	3 ☐	4 ☐
**3.**	0 ☐	1 ☐	2 ☐	3 ☐	4 ☐

Durante os últimos 7 dias:	Não	Raramente	Às vezes	A maior parte do tempo	Sempre
**P3. Você tem se sentido ansioso/a ou preocupado/a com a sua doença ou tratamento?**	0☐	1☐	2☐	3☐	4☐
**P4. Algum dos seus familiares ou amigos ficou ansioso ou preocupado com você?**	0☐	1☐	2☐	3☐	4☐
**P5. Você tem se sentido deprimido/a?**	0☐	1☐	2☐	3☐	4☐

Durante os últimos 7 dias:	*Sim*	*A maior parte do tempo*	*Às vezes*	*Raramente*	*Nunca*
**P6. Você se sentiu em paz?**	0☐	1☐	2☐	3☐	4☐
**P7. Você conseguiu compartilhar com a sua família ou amigos como está se sentido?**	0☐	1☐	2☐	3☐	4☐
**P8. Você tem recebido toda a informação que deseja?**	0☐	1☐	2☐	3☐	4☐

Durante os últimos 7 dias:	Problemas resolvidos ou não tive problemas	Problemas em grande parte resolvidos	Problemas parcialmente resolvidos	Problemas praticamente não resolvidos	Problemas não resolvidos
**P9. Caso tenha enfrentado problemas práticos resultantes da sua doença, eles foram resolvidos (tais como questões pessoais e/ou financeiras)?**	0☐	1☐	2☐	3☐	4☐

Durante os últimos 7 dias:	Nenhum	Até metade de um dia	Mais do metade que a metade de um dia
**P10. Quanto tempo você considera que dedicou a consultas relacionadas com os seus cuidados de saúde (por exemplo, tempo com transportes ou repetição de exames)?**	0☐	1☐	2☐

	Sozinho/a	Com a ajuda de um/a amigo/a ou de um/a familiar	Com a ajuda de um/uma profissional de saúde
**P11. Como você preencheu este questionário?**	0☐	1☐	2☐
