# Supplementary material for: Translation, cross-cultural adaptation, and validation of the Integrated Palliative Care Outcome Scale Renal (IPOS-Renal) for Brazilian Portuguese
Source: J Bras Nefrol. 2026 Jan 16;48(2):e20250133. doi: 10.1590/2175-8239-JBN-2025-0133en (PMC12810525; doi:10.1590/2175-8239-JBN-2025-0133en)
Supplement: Translation

# Translation: Tradução, adaptação transcultural e validação da escala *Integrated Palliative Care Outcome Scale Renal* (IPOS-Renal) para o português do Brasil

**DOI:** 10.1590/2175-8239-JBN-2025-0133pt

**Authors:** Márcia Alvarenga da Trindade, Ana Elizabeth Prado Lima Figueiredo, Carlos Eduardo Poli-de-Figueiredo

**Affiliations:** 1 Pontifícia Universidade Católica do Rio Grande do Sul, Medicina e Ciências da Saúde, Porto Alegre, RS, Brazil.; 2 Pontifícia Universidade Católica do Rio Grande do Sul, Enfermagem, Porto Alegre, RS, Brazil.; 3 Pontifícia Universidade Católica do Rio Grande do Sul, Medicina, Porto Alegre, RS, Brazil.

**Keywords:** Doença renal crônica, doença renal em estágio final, escalas de avaliação, cuidados paliativos, enfermagem

## Abstract

**Introdução:**

A Doença Renal Crônica (DRC) está associada a uma elevada carga de sintomas. No Brasil, não há instrumento específico para aferição de sintomas em pacientes com DRC em cuidados paliativos. A *Integrated Palliative Care Outcome Scale Renal* – (IPOS-Renal) é uma escala já traduzida e validada em treze idiomas.

**Objetivo:**

Traduzir, adaptar transculturalmente e validar a IPOS-Renal para o português do Brasil.

**Métodos:**

Estudo metodológico realizado em oito fases: definição conceitual, tradução para o português, retrotradução, revisão por especialistas, entrevistas cognitivas, leitura de prova, testes psicométricos e relatório final. Foram avaliados 153 pacientes com DRC estágio G5, utilizando a IPOS-Renal e o *Edmonton Symptom Assessment System* (ESAS-r), entre fevereiro e março de 2024, com reaplicação dos instrumentos após quinze dias em 95 pacientes.

**Resultados:**

A escala apresentou consistência interna, confiabilidade, estabilidade temporal e validade convergente com o ESAS-r. Os sintomas mais prevalentes foram sonolência (44%), dificuldade para caminhar (44%), cansaço/falta de energia (43%) e dor (36%). Sintomas menos frequentes incluíram falta de ar (7%), diarreia (14%) e vômitos (14%). A intensidade insuportável foi rara, destacando sintomas emocionais (5%), dificuldade para dormir (3%) e cansaço/falta de energia (3%). A intensidade variou, sendo forte para dor (24–25%) e sonolência (21%), e moderada para dificuldade para caminhar (25%) e cansaço/falta de energia (22%).

**Conclusão:**

A versão em português do Brasil do IPOS-Renal é válida, confiável e adequada para identificar sintomas biopsicossociais em pacientes com DRC estágio 5.

## Introdução

De acordo com a *World Health Organization* ^1^, as doenças renais (DR) ocupam a 9ª posição entre as principais causas de mortalidade global. Entre 2000 e 2022, o número de óbitos relacionados à doença renal crônica (DRC) aumentou significativamente, atingindo 1,3 milhão de pessoas. Projeções indicam que, até 2040, a DRC poderá se tornar a 5ª principal causa de morte no mundo^2^.

O Censo Brasileiro de Diálise de 2024^3^ revelou um aumento de 10% no número estimado de pacientes em diálise em relação a 2023, alcançando 172.585 pessoas e registrando 29.712 óbitos.

Conforme as diretrizes da *Kidney Disease Improving Global Outcome* (KDIGO)*,* a DRC é classificada em seis estágios, de acordo com a taxa de filtração glomerular estimada (TFGe). Nos estágios G1 a G4, adota-se o tratamento conservador, enquanto no estágio G5 — denominado falência renal *(kidney failure)* ou *end-stage kidney disease* (ESKD), indica-se o início da terapia de substituição renal (TSR). Essas orientações são essenciais para o manejo clínico e a otimização dos desfechos^4^.

Os avanços na área da nefrologia têm prolongado a sobrevida de pessoas com DR, o que contribuiu para o aumento da prevalência da DRC entre idosos. Essa população apresenta características clínicas marcadas por múltiplas comorbidades e limitações físicas que comprometem suas condições gerais de saúde. Embora a TSR seja essencial para o manejo da DRC avançada, seus benefícios nem sempre resultam em melhora da qualidade de vida. Pacientes submetidos à dialise, além das limitações inerentes à condição renal, enfrentam desafios relacionados à adaptação às exigências terapêuticas, as quais frequentemente impactam negativamente sua vida^5^.

Ademais, em alguns casos, os pacientes em diálise podem manifestar arrependimento em relação à decisão de iniciar o tratamento. Por isso, é crucial que o plano terapêutico se concentre não apenas na extensão da sobrevida, mas também no bem-estar dos pacientes, incorporando decisões compartilhadas com base em informações claras e que considerem seus valores, expectativas e necessidades específicas^6^.

Neste contexto, os cuidados paliativos são amplamente recomendados, pois sua abordagem interdisciplinar considera a biografia, os valores e os objetivos pessoais do paciente, colocando-o no centro do cuidado^7^.

Os cuidados paliativos têm como objetivo melhorar a qualidade de vida de pacientes com doenças crônicas e ameaçadoras da vida, aliviando o sofrimento por meio da identificação e do manejo adequados dos sintomas, além de oferecer suporte psicológico, social e espiritual tanto aos pacientes quanto aos familiares. Tal abordagem fortalece o vínculo com o paciente, possibilitando o planejamento antecipado do cuidado e a participação ativa nas decisões terapêuticas ao longo do tratamento. Destaca-se a importância das equipes multiprofissionais no acompanhamento do paciente com DRC avançada, auxiliando no planejamento de estratégias para um cuidado integral^8^.

A *Palliative Care Quality Network* analisou uma coorte com 33.183 pacientes internados, todos submetidos à consulta de cuidados paliativos durante a hospitalização. Foram comparados 02 grupos: pacientes com DRC, totalizando 1.057 indivíduos (3,2%), e pacientes com outras doenças graves, correspondendo ao restante da coorte. Ambos os grupos apresentavam características clínicas semelhantes, incluindo baixa pontuação de performance (~35%), ansiedade moderada a grave (~15%) e náusea (6%). Após a consulta, observou-se melhora geral dos sintomas, com redução mais expressiva da ansiedade no grupo de pacientes com DRC (92% versus 66%; p = 0,002). Entretanto, esse grupo foi encaminhado com menor frequência aos programas de cuidados paliativos em comparação aos pacientes com outras doenças graves (30,7% versus 37,6%; p < 0,001). Apesar da alta morbidade e mortalidade associada à DRC avançada, esses pacientes são submetidos a cuidados paliativos com menor regularidade, possivelmente em decorrência da percepção equivocada quanto à necessidade desses serviços^9^.

Muitos profissionais têm dúvidas sobre o encaminhamento de pacientes para cuidados paliativos. Para auxiliar nesse processo de elegibilidade, a Universidade de Edimburgo elaborou o *Supportive and Palliative Care Indicators Tool* (SPICT), uma ferramenta destinada a identificar sinais de necessidade de cuidados paliativos em pacientes com doenças avançadas. O SPICT-BR (versão brasileira) identifica pessoas em processo de deterioração da saúde, incluindo indicadores específicos para a DRC, como os estágios G4 e G5, que se associam à piora clínica. Além disso, considera a DR como fator complicador em outras condições limitantes ou tratamentos e aborda a discussão sobre a decisão de não realizar diálise em casos de piora clínica ou intolerância ao tratamento. Dessa forma, o SPICT-BR oferece uma abordagem mais precisa e sensível às necessidades específicas de pacientes com DRC avançada, permitindo intervenções oportunas e direcionadas aos cuidados paliativos^10^.

Destaca-se a necessidade de instrumentos específicos para avaliar e planejar o cuidado, permitindo uma investigação eficaz das necessidades dos indivíduos. No contexto dos cuidados paliativos, o *Edmonton Symptoms Assesment System* (ESAS-r)^11^ é amplamente utilizado para mensurar a intensidade dos sintomas. Essa escala classifica os sintomas como leves (0 a 3), moderados (4 a 7) e intensos (8 a 10), auxiliando na análise da complexidade clínica. Além disso, fornece uma base sólida para o desenvolvimento de estratégias de intervenção individualizadas^12^.

A perda da funcionalidade é uma característica marcante em pacientes sob cuidados paliativos^13^. Nesse contexto, a avaliação do desempenho funcional é essencial para estimar o prognóstico do paciente. A *Palliative Performance Scale* (PPS) avalia mobilidade, atividade e evidências de doença, autocuidado, ingesta alimentar e estado de consciência, atribuindo valores entre 0% (morte) e 100% (funcionalidade plena). Além de oferecer um panorama clínico, essa escala pode integrar avalições prognósticas mais abrangentes^14^.

Contudo, tanto a PPS quanto o ESAS-r não são escalas específicas para avaliar sintomas em pacientes com DRC em cuidados paliativos e, até o momento, não há no Brasil um instrumento direcionado a essa população. No cenário internacional, o *Cicely Saunders Institute, do King’s College London* (KCL-CSI), desenvolveu a *Integrated Palliative Care Outcome Scale Renal* (IPOS-Renal), um instrumento específico para a avaliação integral dos sintomas em pacientes com DRC avançada em cuidados paliativos. Essa escala já foi traduzida e adaptada transculturalmente para treze idiomas^15^.

Dessa forma, este estudo teve como objetivo traduzir, validar e adaptar transculturalmente a IPOS-Renal para o português do Brasil.

## Métodos

O estudo caracterizou-se como metodológico, com o objetivo de traduzir, validar e adaptar transculturalmente a IPOS-Renal para o português do Brasil.

Os autores seguiram as diretrizes estabelecidas pelo KCL-CSI para garantir a efetividade do processo. O estudo foi conduzido em oito fases, descritas a seguir:

**Fase I – Definição conceitual:**

A definição conceitual foi realizada em três etapas:

I) Revisão da literatura sobre a qualidade de vida de pacientes em cuidados paliativos.
II) Reunião com sete profissionais especialistas em cuidados paliativos, sendo dois médicos, três psicólogos, uma enfermeira e um assistente espiritual. O grupo avaliou e discutiu cada item do IPOS-Renal, estabelecendo consenso sobre sua adequação e relevância para a prática clínica.
III) Por meio de dois grupos de pacientes com DRC avançada, investigou-se se a versão do IPOS-Renal definida na Fase II seria reconhecida pelos doentes. Foram selecionados três pacientes em hemodiálise e dois em diálise peritoneal, ajustando a IPOS-Renal a partir das sugestões dos participantes.

**Fase II – Tradução direta para o português do Brasil (com dois tradutores e um mediador):**

Nesta fase, dois tradutores nativos do Brasil realizaram, de forma independente, a tradução do IPOS-Renal original (em inglês) para o português. Em seguida, um mediador recebeu as duas versões e as sintetizou em um único documento.

**Fase III (cega) – Tradução reversa do português para o inglês:**

Dois tradutores nativos de língua inglesa realizaram a retrotradução do documento em português produzido na Fase II, sem acesso à versão original. Após essa etapa, o mediador foi novamente envolvido, recebendo as duas traduções e sintetizando-as em um único documento.

**Fase IV – Revisão dos especialistas:**

Formou-se um grupo composto por todos os envolvidos no processo: profissionais de saúde, tradutores, mediador e pesquisadora. Após amplas discussões, alcançou-se consenso sobre a versão pré-final do IPOS-Renal, que foi então enviada ao KCL-CSI para revisão. O KCL-CSI sugeriu alguns ajustes, os quais foram discutidos e alinhados entre os pesquisadores. Em seguida, o documento foi reenviado para reavaliação pelo KCL-CSI, com o objetivo de obter aprovação da versão pré-final.

**Fase V – Entrevista cognitiva – pré-teste do IPOS-Renal no Brasil:**

Nesta fase, foi realizado o pré-teste do IPOS-Renal, envolvendo dez pacientes em estágio G5 e sete profissionais de saúde. O objetivo foi avaliar a compreensão dos participantes em relação aos itens do instrumento. Todos os indivíduos demonstraram plena compreensão de cada item, não havendo necessidade de ajustes para a versão final.

**Fase VI – Leitura da prova:**

A versão final do IPOS-Renal foi encaminhada ao KCL-CSI para aprovação e autorização do início dos testes psicométricos.

**Fase VII – Testes psicométricos:**

Para este estudo, foram estabelecidos como critérios de inclusão: pacientes adultos, com idade igual ou superior a 18 anos, falantes nativos do português, residentes no Brasil e com diagnóstico de DRC avançada (estágio G5) em tratamento com hemodiálise ou diálise peritoneal.

Os critérios de exclusão foram: pacientes com dificuldades de comunicação, limitações físicas que os tornassem incapazes de participar da coleta de dados ou expectativa de óbito em poucos dias. Para a avaliação cognitiva, aplicou-se o Miniexame do Estado Mental, sendo excluídos aqueles que apresentaram déficit cognitivo. Além disso, foi respeitada a decisão dos pacientes que recusaram participar do estudo^16^.

Os pacientes foram avaliados utilizando o novo instrumento IPOS-Renal e, para a análise de validade convergente, empregou-se a escala ESAS-r como variável externa. Utilizou-se o coeficiente de correlação, considerando que ambas as escalas mensuram o impacto e a intensidade dos sintomas.

A coleta de dados foi realizada entre fevereiro e março de 2024, com a inclusão de 153 pacientes com DRC avançada estágio G5, sendo 139 em hemodiálise e 14 em diálise peritoneal. Em 95 desses pacientes, os instrumentos foram reaplicados após um intervalo de quinze dias.

**Fase VIII – Relatório e publicação:**

A versão final do IPOS-Renal em português do Brasil foi oficialmente publicada no site do KCL-CSI e encontra-se disponível para acesso público e aplicação clínica.

### Aspectos Éticos

Os pesquisadores seguiram os princípios éticos e legais estabelecidos pelas Normas e Diretrizes Regulamentadoras para Pesquisa com Seres Humanos^17^. O estudo foi aprovado pelo Comitê de Ética em Pesquisa da PUCRS, sob o parecer nº 6.329.294. Todos os participantes assinaram o Termo de Consentimento Livre e Esclarecido, enquanto os pesquisadores assinaram o Termo de Compromisso para Utilização dos Dados, comprometendo-se a utilizar as informações exclusivamente para fins de pesquisa, mantendo o sigilo e o anonimato de todos os participantes.

### Análise Estatística

Os dados foram organizados em planilha eletrônica no Microsoft Excel® e analisados no programa SPSS, versão 25.0, adotando-se nível de significância de 5%. Foram aplicadas estatísticas descritivas (frequências absoluta e relativa, médias, medianas, desvios-padrão, amplitude e amplitude interquartílica). A normalidade das variáveis contínuas foi testada pelo método *Kolmogorov-Smirnov*. A confiabilidade do instrumento foi avaliada pelo coeficiente alfa de *Cronbach* (α), considerando-se aceitáveis valores entre 0,70 e 0,95. A correlação entre variáveis contínuas foi examinada pelos coeficientes de *Pearson* ou *Spearman* (quando distribuição assimétrica), classificados como muito fraca (0–0,199), fraca (0,200–0,399), moderada (0,400–0,699), forte (0,700–0,899) e muito forte (0,900–1,0).

## Resultados

Os resultados apresentados referem-se a uma amostra de 153 pacientes com DRC avançada estágio G5. Os pacientes foram avaliados por meio de um questionário que incluiu dados sociodemográficos e a aplicação das escalas ESAS-r e IPOS-Renal. Os resultados referentes à caracterização da amostra estão descritos na Tabela 1 abaixo:

**Tabela 1:** Caracterização geral da amostra (N = 153)

Variável	n	%	Média	Desvio	Amplitude
**Sexo**					
Masculino	93	60,8			
Feminino	59	38,6			
Outro	1	0,6			
Idade (anos)			57,8	13,3	24–82
Até 29 anos	5	3,3			
De 30 a 39	12	7,8			
De 40 a 49	23	15,0			
De 50 a 59	36	23,5			
De 60 a 69	44	28,8			
Setenta ou mais	33	21,6			
**Estado civil**					
Solteiro(a)	27	17,8			
Casado(a)	72	47,3			
União estável	21	13,2			
Divorciado(a)	17	11,2			
Viúvo(a)	16	10,5			
**Filhos**					
Nenhum	38	26,0			
De 1 a 2	69	45,0			
De 3 a 4	33	22,0			
Cinco ou mais	13	7,0			
**Escolaridade**					
Analfabeto	2	1,3			
Ensino fundamental completo	10	6,5			
Ensino fundamental incompleto	40	26,1			
Ensino médio completo	39	25,5			
Ensino médio incompleto	13	8,5			
Curso superior completo	26	17,0			
Curso superior incompleto	8	5,2			
Pós-graduação completa	9	5,9			
Pós-graduação em andamento	1	0,7			
Mestrado	3	2,0			
Doutorado	2	1,3			
**Profissão**					
Especialistas, profissões intelectuais/científicas	38	24,8			
Técnicos e profissões de nível médio	28	18,3			
Pessoal administrativo e similares	33	21,6			
Operários, artífices, trabalhadores similares	28	18,3			
Do lar	12	7,8			
Outros	14	9,2			
**Situação profissional**					
Empregado(a)	18	11,8			
Desempregado(a)	2	1,3			
Aposentado(a)	90	58,8			
Licença saúde	29	19,0			
Autônomo(a)	14	9,1			
**Onde mora**					
Interior do RS	7	4,6			
Litoral	3	2,0			
Outro Estado	1	0,7			
Porto Alegre	104	68,0			
Região Metropolitana de Porto Alegre	38	24,7			
**Tipo de domicílio**					
Casa	107	69,9			
Apartamento	45	29,4			
Instituição de Longa Permanência	1	0,7			
**Número de pessoas com quem reside**					
Nenhuma	22	14,4			
Uma	57	37,3			
Duas	38	24,8			
Três	21	13,7			
Quatro ou mais	15	9,8			
**Possui religião**					
Sim	134	89,3			
Não	19	10,7			
**Crença ou religião**					
Acredita em Deus/Jesus Cristo	4	2,6			
Adventista	3	2,0			
Budista	1	0,7			
Católica	77	50,3			
Católico/Espírita Kardecista	1	0,7			
Cristão	7	4,6			
Kardecista, Candomblé, Nação e igreja Católica	1	0,7			
Espiritualidade, Espírita Kardecista	15	9,8			
Evangélica	14	9,1			
Matriz Africana	12	7,7			
*Seicho-No-Ie*	2	1,3			
Testemunha de Jeová	3	2,0			
Não tem religião e não crê em nada	13	8,5			
**Tempo de terapia (anos)**			3,5	3,6	15 dias–22 anos
Até 6 meses	28	18,3			
Mais de 6 meses a 1 ano	16	10,5			
Mais de 1 ano a 2 anos	29	19,0			
Mais de 2 anos a 5 anos	47	30,7			
Mais de 5 anos	33	21,5			
**Tipo de acesso vascular**					
Prótese	3	2,0			
Fístula Arteriovenosa	81	52,9			
Cateter Venoso Central	55	35,9			
Cateter Peritoneal	14	9,2			
**Doenças**					
Hipertensão Arterial Sistêmica	121	79,8			
Diabetes Mellitus	32	20,2			
**Número de outras doenças apontadas**					
Uma	26	17,0			
Duas a três	57	37,2			
Quatro a cinco	43	28,1			
Seis ou mais	27	17,7			

Notas – A: Percentuais obtidos com base no total da amostra. Valores apresentados como n (%) salvo indicação em contrário. Para variáveis contínuas, apresentam-se média, desvio-padrão (DP) e amplitude. Percentuais calculados com base no total da amostra (n = 153).

A amostra foi composta majoritariamente por homens (60,8%), com média de idade de 57,8 anos (DP = 13,3), predominando as faixas etárias de 60 a 69 anos (28,8%) e de 50 a 59 anos (23,5%). A maioria era casada (47,4%), possuía de 1 a 2 filhos (42,1%) e apresentava escolaridade até o ensino fundamental ou médio (ambos com 34,0%). Profissões intelectuais/científicas (24,8%) e funções administrativas (21,6%) foram as mais frequentes, com predomínio de aposentados (58,8%). Quanto à moradia, 68,0% residiam em Porto Alegre e 69,9% viviam em casas; 37,3% moravam sozinhos e 24,8% com outra pessoa. A maioria declarou ter religião (89,3%), predominantemente católica (50,3%). O tempo de hemodiálise ou diálise peritoneal mais comum foi de 2 a 5 anos (30,7%), seguido de >5 anos (21,6%). O acesso vascular predominante foi a fístula arteriovenosa (52,9%), seguido do cateter venoso central (35,9%). Entre as comorbidades, destacaram-se a hipertensão arterial (87,6%) e o diabetes mellitus (40,5%). O número de outras doenças além da DRC variou, sendo mais frequente de 2 a 3 (35,6%), enquanto 19,3% relataram ≥ 6.

Os resultados do IPOS-Renal na primeira avaliação estão apresentados na Tabela 2 a seguir:

**Tabela 2:** Distribuição absoluta e relativa dos itens do ipos-renal na primeira avaliação (n = 153)

Sintomas			Classificações da escala IPOS-Renal									
	Prevalência		Não (0)		Leve (1)		Moderado (2)		Forte (3)		Insuportável (4)	
	n	%	n	%	n	%	n	%	n	%	n	%
**Sintomas referidos na escala**												
Dor	55	35,9	88	57,5	10	6,5	15	9,8	36	23,5	4	2,6
Falta de ar	10	6,6	130	85,0	13	8,5	5	3,3	5	3,3		
Cansaço/falta de energia	65	42,5	55	35,9	33	21,6	34	22,2	26	17,0	5	3,3
Náuseas/enjoo	34	22,2	102	66,7	17	11,1	15	9,8	15	9,8	4	2,6
Vômitos	22	14,3	123	80,4	8	5,2	8	5,2	10	6,5	4	2,6
Pouco apetite	40	26,2	103	67,3	10	6,5	24	15,7	15	9,8	1	0,7
Intestino preso	27	17,6	117	76,5	9	5,9	12	7,8	11	7,2	4	2,6
Feridas na boca/boca seca	24	15,7	118	77,1	11	7,2	13	8,5	8	5,2	3	2,0
Sonolência	67	43,8	63	41,2	23	15,0	32	20,9	32	20,9	3	2,0
Dificuldade para caminhar	67	43,8	68	45,6	18	11,8	38	24,8	24	15,7	5	3,3
Coceira	33	21,5	94	61,3	26	17,0	19	12,4	12	7,8	2	1,3
Dificuldade para dormir	49	32,1	81	52,9	23	15,0	24	15,7	20	13,1	5	3,3
Pernas inquietas	25	16,4	106	69,3	22	14,4	15	9,8	9	5,9	1	0,7
Alterações na pele	28	18,3	105	68,6	20	13,1	15	9,8	10	6,5	3	2,0
Diarreia	21	13,7	124	81,0	8	5,2	13	8,5	8	5,2	0	0,0
**Sintomas extras referidos em questão aberta**												
Dores/local da dor	65	42,5	88	57,5	7	4,6	19	12,4	38	24,8	1	0,7
Sintomas emocionais	25	16,3	128	83,7	1	0,7	1	0,7	16	10,5	7	4,6
Sintomas relacionados à pressão arterial e volemia	13	8,5	140	91,5	2	1,3	1	0,7	10	6,5	0	0,0
Sintomas do trato gastrointestinal	16	10,5	137	89,5	3	2,0	1	0,7	9	5,9	3	2,0
Durante os últimos 7 dias			Não (0)		Raramente (1)		Às vezes (2)		A maior parte do tempo (3)		Sempre (4)	
P3. Você tem se sentido ansioso(a) ou preocupado(a) com a sua doença ou tratamento?	99	64,7	54	35,3			43	28,1	18	11,8	38	24,8
P4. Algum dos seus familiares ou amigos ficou ansioso ou preocupado com você?	134	87,6	13	8,5	6	3,9	28	18,3	29	19,0	77	50,3
P5. Você tem se sentido deprimido(a)?	44	28,8	100	65,4	9	5,9	27	17,6	3	2,0	14	9,2
Durante os últimos 7 dias			Sim (0)		A maior parte do tempo (1)		Às vezes (2)		Raramente (3)		Nunca (4)	
P6. Você se sentiu em paz?	46	30,0	87	56,9	20	13,1	17	11,1	2	1,3	27	17,6
P7. Você conseguiu compartilhar com a sua família ou amigos como está se sentindo?	65	42,5	65	42,5	23	15,0	20	13,1	6	3,9	39	25,5
P8. Você tem recebido toda a informação que deseja?	22	14,4	122	79,7	9	5,9	15	9,8	4	2,6	3	2,0
Durante os últimos 7 dias			Problemas resolvidos ou não tive problemas (0)		Problemas em grande parte resolvidos (1)		Problemas parcialmente resolvidos (2)		Problemas praticamente não resolvidos (3)		Problemas não resolvidos (4)	
P9. Caso tenha enfrentado problemas práticos resultantes da sua doença, eles foram resolvidos (tais como questões pessoais e/ou financeiras)?	63	41,2	71	46,4	19	12,4	52	34,0	4	2,6	7	4,6
Durante os últimos 7 dias			Nenhum (0)		Até metade de um dia (1)		Mais do que a metade de um dia (2)					
P10. Quanto tempo você considera que dedicou a consultas relacionadas aos seus cuidados de saúde (por exemplo, tempo com transporte ou repetição de exames)?					65	42,5	88	57,5				
Durante os últimos 7 dias			Sozinho(a) (0)		Com a ajuda de um(a) amigo(a) ou de um familiar (0)		Com a ajuda de um(a) profissional de saúde (153)					
P11. Como você preencheu este questionário?								100%				

Notas – Prevalência definida como sendo referente a sintomas classificados como moderados, fortes e insuportáveis.

Após a aplicação da escala IPOS-Renal, os sintomas mais prevalentes foram sonolência 43,8% (n = 67), dificuldade para caminhar 43,8% (n = 67), cansaço/falta de energia 42,5% (n = 65) e dor 35,9% (n = 55). Os menos frequentes foram falta de ar 6,6% (n = 10), diarreia 13,7% (n = 21) e vômitos 14,3% (n = 22).

Na intensidade insuportável, os maiores percentuais foram de sintomas emocionais 4,6% (n = 7), dificuldade para dormir 3,3% (n = 5) e cansaço/falta de energia 3,3% (n = 5).

Entre os classificados como fortes, predominaram dor/local da dor (queixa específica do local da dor) 24,8% (n = 38), dor 23,5% (n = 36) e sonolência 20,9% (n = 32).

Nos casos avaliados como moderados, as maiores frequências foram dificuldade para caminhar 24,8% (n = 38), cansaço/falta de energia 22,2% (n = 34) e sonolência 20,9% (n = 32).

A ausência de sintomas foi observada principalmente para falta de ar 85,0% (n = 130), vômitos 80,4% (n = 123) e diarreia 81,0% (n = 124).

Nas seções referentes ao período “durante os últimos 7 dias”, aproximadamente metade da amostra, 50,3% (n = 77), relatou que sempre há algum familiar ou amigo ansioso ou preocupado com o paciente. Por outro lado, 65,4% (n = 100) afirmaram não apresentar sintomas de depressão. Em relação à sensação de paz, 56,9% (n = 87) declararam sentir-se em paz, e 79,7% (n = 122) relataram ter recebido todas as informações que desejavam.

Quanto ao enfrentamento de problemas práticos decorrentes da doença, a maioria, 46,7% (n = 71), relatou não ter enfrentado problemas a serem resolvidos, enquanto 34,0% (n = 52) indicaram que os problemas foram parcialmente solucionados. No que se refere ao tempo dedicado às consultasmédicas, à espera, à realização ou repetição de exames e ao deslocamento, 57,5% (n = 88) dos participantes relataram investir mais de metade de um dia nessas atividades.

Na análise estatística, a nova versão do IPOS-Renal foi testada quanto à consistência interna, reprodutibilidade (teste-reteste), validade de construto, validade de critério (validade concorrente e/ou preditiva) e capacidade de resposta à mudança. O coeficiente alfa de *Cronbach* apresentou consistência interna e confiabilidade satisfatórias, demonstrando coerência entre os itens da escala. As consistências internas mais baixas apresentaram confiabilidade aceitável, conforme demonstrado na Tabela 3.

**Tabela 3:** Análise de consistência interna pelo coeficiente alfa de *cronbach* (se item excluído): ipos-renal (n = 153)

Sintomas	Média de escala se o item for excluído	Variância de escala se o item for excluído	Correlação de item total corrigida	Correlação múltipla ao quadrado	Alfa de *Cronbach* se o item for excluído
P2_Dor	10,95	45,484	0,415	0,524	0,799
P2_Falta_de_ar	11,77	51,559	0,307	0,282	0,703
P2_Cansaço_falta_de_energia	10,72	46,467	0,424	0,327	0,700
P2_Náuseas_enjoo	11,31	46,993	0,424	0,571	0,701
P2_Vômitos	11,56	47,814	0,426	0,587	0,704
P2_Pouco_apetite	11,32	51,416	0,260	0,234	0,677
P2_Intestino_preso	11,48	54,580	0,360	0,275	0,698
P2_Feridas_na_boca_ou_boca_seca	11,54	51,355	0,386	0,247	0,702
P2_Dificuldade_para_caminhar	10,80	46,211	0,420	0,340	0,722
P2_Coceira	11,31	51,783	0,139	0,227	0,678
P2_Dificuldade_para_dormir_	11,03	47,742	0,336	0,271	0,713
P2_Pernas_inquietas_	11,48	50,225	0,290	0,283	0,721
P2_Alterações_na_pele	11,42	49,653	0,291	0,210	0,690
P2_Diarreia	11,64	52,258	0,161	0,211	0,692
Sintoma1_Dor_local	10,95	49,294	0,210	0,454	0,732
Sintoma2_Sintomas_Emocionais	11,50	51,712	0,204	0,166	0,645
Sintoma3_Pressão_Arterial_Volemia	11,80	52,978	0,127	0,230	0,737
Sintoma4_Gastrointestinal	11,73	52,895	0,196	0,143	0,641

Notas – Valores de alfa de *Cronbach* ≥ 0,70 indicam consistência interna satisfatória; valores entre 0,60 e 0,69 podem ser considerados aceitáveis em estudos exploratórios.

O tempo médio de reaplicação entre o teste e o reteste foi de quinze dias. A avaliação revelou correlações positivas, classificadas como fracas (|0,200| a |0,399|) a moderadas (|0,400| a |0,699|). As estimativas mais elevadas foram observadas nos sintomas pouco apetite (r = 0,677), dificuldade para caminhar (r = 0,676) e dificuldade para dormir (r = 0,681). Em contrapartida, as menores correlações ocorreram nos sintomas emocionais (r = 0,288) e falta de ar (r = 0,328).

O ESAS-r é um questionário composto por nove sintomas e uma décima opção adicional. As pontuações de apetite e bem-estar foram revertidas. As médias dos sintomas variaram de 0,60 (DP = 1,89) para náusea a 3,03 (DP = 2,41) para bem-estar, sendo a maioria dos sintomas de intensidade leve, com maiores escores para sonolência, apetite e bem-estar. No item destinado a sintomas adicionais, foram relatadas queixas de dor/local da dor, sintomas gastrointestinais e questões emocionais.

O escore médio total foi de 27,8 (DP = 10,2). No entanto, a pontuação total da escala no teste-reteste não apresentou diferenças significativas (p = 0,180), indicando estabilidade entre as pontuações médias das avaliações inicial (26,6 ± 9,8) e final (28,4 ± 11,1). Os dados referentes à escala ESAS-r estão apresentados na Tabela 4.

**Tabela 4:** Média, desvio-padrão e mediana dos itens da escala esas-r, na avaliação inicial e no reteste (n = 95)

Itens	Escala ESAS-r (n = 95)						
	Avaliação inicial			Avaliação final			
	Média	Desvio-padrão	Mediana	Média	Desvio-padrão	Mediana	p^B^
ESAS_1Dor	0,91	2,15	0,00	1,60	2,66	0,00	0,234
ESAS_2Cansaço	1,36	2,96	0,00	2,20	3,12	0,00	0,014
ESAS_3Sonolência	2,78	3,25	0,00	2,87	3,38	0,00	0,828
ESAS_4Náusea	0,55	1,73	0,00	0,32	1,44	0,00	0,310
ESAS_5COM Apetite_REVERSA	2,60	3,11	1,00	3,18	2,12	2,00	0,259
ESAS_6Falta_ar	0,11	0,72	0,00	0,25	1,30	0,00	0,343
ESAS_7Depressão	1,24	2,62	0,00	1,39	2,75	0,00	0,629
ESAS_8Ansiedade	2,58	3,25	0,00	2,14	3,25	0,00	0,260
ESAS_9Bem_estar_ REVERSA	2,93	3,03	2,00	2,64	2,68	2,00	0,068
ESAS_10Outros^F^	1,98	2,01	2,00	2,06	1,96	2,00	0,075
ESAS_Total	26,6	9,8	24,0	28,4	11,1	28,0	0,180

Notas – B: Teste de *Wilcoxon*. F: Outros – Dor/local da dor, sintomas emocionais e do trato gastrointestinal.

Para a análise de validade convergente, utilizou-se o ESAS-r como variável externa. Considerou-se o coeficiente de correlação, uma vez que ambas as escalas avaliam o impacto e a intensidade dos sintomas. Os resultados estão apresentados na Tabela 5.

**Tabela 5:** Validade convergente entre ipos-renal e esas-r por meio do coeficiente de correlação de *spearman*

Sintomas	Coeficiente de correlação de *Spearman* (r_S_) – ESAS-r										
	Dor	Cansaço	Sonolência	Náusea	COM Apetite REVERSA	Falta ar	Depressão	Ansiedade	Bem-estar REVERSA	Outros	Total
P2_Dor	0,417**	0,098	-0,075	-0,014	-0,027	0,076	0,172*	0,194*	-0,068	0,220**	0,315**
P2_Falta_de_ar	0,114	0,237**	0,171*	0,056	-0,037	0,212**	0,050	0,177*	-0,108	0,205*	0,398*
P2_Cansaço_falta_de_energia	0,222**	0,403**	-0,018	0,060	0,019	0,110	0,213**	0,243**	-0,141	0,305**	0,489**
P2_Náuseas_enjoo	0,008	0,177*	-0,009	0,185*	-0,197*	-0,068	-0,035	0,014	0,088	0,032	0,061
P2_Vômitos	0,044	0,227**	-0,074	0,247**	-0,111	-0,090	0,050	0,165*	0,059	0,158	0,265*
P2_Pouco_apetite	0,030	0,071	0,100	0,067	0,009	0,207*	0,177*	0,012	-0,130	0,146	0,151
P2_Intestino_preso	-0,035	0,051	0,055	-0,004	0,041	0,172*	0,016	0,037	0,005	0,092	0,065
P2_Feridas_na_boca_ou_boca_seca	-0,025	0,030	0,037	0,064	-0,105	0,074	0,104	0,173*	-0,007	0,149	0,111
P2_Dificuldade_para_caminhar	0,163*	0,108	0,003	0,168*	-0,002	0,166*	0,205*	0,215**	0,017	0,299**	0,502**
P2_Coceira	-0,037	-0,012	-0,123	-0,050	-0,097	-0,141	-0,048	-0,078	0,123	-0,032	-0,080
P2_Dificuldade_para_dormir	0,043	0,068	-0,070	0,077	-0,033	0,096	0,116	0,189*	-0,046	0,152	0,137
P2_Pernas_inquietas	-0,050	0,090	-0,085	0,032	-0,052	-0,036	-0,076	0,042	-0,011	0,016	-0,010
P2_Alterações_na_pele	0,022	0,164*	-0,053	0,052	-0,050	0,160*	0,090	0,140	-0,107	0,148	0,120
P2_Diarreia	0,117	0,000	-0,027	-0,012	-0,025	0,124	0,112	0,199*	0,000	0,076	0,129
Sintoma1_Dor_local	0,388**	0,049	-0,060	-0,003	0,049	0,059	0,132	0,111	-0,031	0,104	0,156
Sintoma2_Sintomas_Emocionais	0,071	0,018	-0,045	0,001	-0,073	0,238**	-0,003	0,060	0,196*	0,100	0,129
Sintoma3_Pressão_Arterial_Volemia	0,084	0,192*	-0,040	0,253**	0,054	-0,056	0,191*	0,104	-0,034	0,166*	0,389*
Sintoma4_Gastrointestinal	0,083	0,032	-0,093	-0,050	0,063	0,169*	0,060	-0,108	0,016	0,035	0,007

Notas – *Significância a 5% (p < 0,05).

**Significância a 1% (p < 0,001).

Para o estudo de validade convergente da IPOS-Renal, utilizou-se a ESAS como variável externa, considerando-se o coeficiente de correlação entre os escores das duas escalas. Conforme apresentado na Tabela 5, observaram-se correlações positivas significativas entre o escore total da ESAS-r e os sintomas da IPOS-Renal, classificadas como moderadas para cansaço/falta de energia (r = 0,489; p < 0,01) e dificuldade para caminhar (r = 0,502; p < 0,001). Também foram verificadas correlações significativas fracas, porém relevantes, com dor (r = 0,315; p < 0,001), falta de ar (r = 0,398; p < 0,01), sintomas relacionados à pressão arterial/volemia (r = 0,389; p < 0,001) e vômitos (r = 0,265; p < 0,05). Esses resultados fornecem evidências de validade convergente da IPOS-Renal nesta amostra.

## Discussão

A DRC impõe uma carga elevada de sintomas, muitas vezes difíceis de aliviar com recursos convencionais. Profissionais de saúde podem apresentar dificuldades em reconhecer determinados sintomas, o que resulta na persistência de queixas não tratadas. Assim, a identificação precoce e a implementação de estratégias adequadas são essenciais para minimizar o sofrimento^18^.

Apesar da importância da avaliação rotineira dos sintomas, ainda existem lacunas na prática clínica. Mesmo quando relatados, os tratamentos disponíveis são limitados e, muitas vezes, não são iniciados, seja pela escassez de evidências específicas para essa população ou pela complexidade das interações medicamentosas na doença renal. No entanto, cabe aos profissionais de saúde realizar uma avaliação criteriosa e oferecer cuidados individualizados de forma eficaz^19^.

Neste estudo, a aplicação do IPOS-Renal evidenciou como sintomas prevalentes a sonolência, a dificuldade para caminhar, o cansaço, a falta de energia e a dor. Esses achados são consistentes com a validação do IPOS-Renal em Portugal^20^, que também identificou dor (23,8%), pouco apetite (23,1%), dificuldade para dormir (17,9%) e cansaço/falta de energia (17,1%). Além disso, corroboram estudos prévios que destacam a frequência e o impacto desses sintomas em indivíduos com DRC avançada^21,22^.

Ademais, investigou-se a percepção dos pacientes sobre o tempo dedicado a consultas, exames, transporte e deslocamentos para o tratamento. A maioria revelou que esse tempo ocupa mais da metade do dia, exigindo descanso ao chegar em casa. Alguns mencionaram a necessidade de dormir, reforçando a alta prevalência de fadiga, cansaço/falta de energia e sonolência, bem como o impacto desses sintomas na rotina diária^23^.

Um estudo recente realizado no Brasil corrobora os achados deste trabalho. A pesquisa analisou o impacto da fadiga no grau de dependência de pacientes em hemodiálise e constatou que 63 dos 101 participantes (62,4%) apresentavam fadiga pós-diálise. Essa condição esteve associada ao tempo de hemodiálise (p = 0,041), ao tempo de recuperação após as sessões (p < 0,001) e ao turno da sessão (p < 0,001), sendo menos frequente entre aqueles que realizavam o tratamento à noite^24^. Esses dados convergem com os relatos dos pacientes entrevistados, que apontaram o aproveitamento do sono noturno, após diálise noturna, como forma de recuperação do cansaço e da falta de energia. Em contraste, aqueles que realizavam o tratamento durante o dia relataram a necessidade de descanso ao chegarem em casa, o que pode ter contribuído para as queixas de dificuldade para dormir à noite e sonolência diurna.

Na Dinamarca, a aplicação do IPOS-Renal revelou a prevalência de queixas de fadiga e redução da mobilidade, em consonância com os achados deste estudo. Além disso, os participantes relataram tonturas, alterações de memória e tristeza associada à dependência de familiares e profissionais de saúde, fatores que contribuíram para o isolamento social e a perda da identidade^22-25^.

A presente pesquisa evidenciou sintomas na esfera emocional, com 0,7% das queixas classificadas como leves, 0,7% como moderadas, 10,5% como fortes e 4,6% como insuportáveis. A presença de sintomas emocionais em pacientes em diálise foi confirmada por outros autores, que destacam a importância da identificação precoce. Depressão e ansiedade, associadas à renda familiar, impactam negativamente o cotidiano desses pacientes^26,27^.

Em uma pesquisa que analisou a influência dos sintomas psicopatológicos no diagnóstico, prognóstico e resposta ao tratamento da insuficiência renal, a aplicação da escala de alexitimia revelou escores elevados, evidenciando sua associação a esses sintomas^28^. O termo “alexitimia” foi introduzido em 1972 para descrever pacientes com dificuldades na expressão de seus afetos, sendo composto pelo prefixo grego *a*, que significa “privação”, e *lexis*, que se refere a *thymos*, ou “humor”^29^. No presente estudo, observou-se que os pacientes apresentaram dificuldades em nomear seus sentimentos em relação às mudanças decorrentes do tratamento dialítico. Embora negassem queixas de depressão (65,4%) e afirmassem sentir-se em paz (56,9%), essa negação contrasta com a angústia manifestada quando questionados sobre suas condições de saúde.

A primeira pergunta do questionário IPOS-Renal é aberta, permitindo que os pacientes respondam livremente sobre seus principais problemas ou preocupações dos últimos sete dias, podendo indicar até três respostas. A partir dos relatos mais frequentes, emergiram três categorias: (1) preocupação com a própria saúde de maneira geral, incluindo sintomas e outras comorbidades, além da DRC; (2) preocupações financeiras; e (3) preocupações com familiares e a saúde destes.

Em relação à preocupação com a própria saúde, os pacientes expressaram ansiedade, especialmente entre aqueles que aguardam um transplante. Essa ansiedade manifesta-se na constante expectativa de receber uma ligação informando sobre a disponibilidade de um órgão, bem como na incerteza quanto às condições clínicas necessárias para a inclusão na lista de espera para o procedimento. Além disso, pacientes que já foram transplantados, mas perderam o enxerto e retornaram à diálise, vivenciam angústia diante do temor da ineficácia do tratamento, sobretudo ao testemunharem óbitos de outros pacientes na mesma condição durante as sessões de diálise.

Esses achados evidenciam a dualidade entre a percepção de bem-estar e as preocupações subjacentes, destacando a necessidade de uma abordagem integrada que contemple tanto os aspectos físicos quanto os emocionais no tratamento da DRC avançada. Essa perspectiva é fundamental para promover uma melhor qualidade de vida aos pacientes, considerando as complexidades emocionais que permeiam sua experiência ao longo da trajetória do tratamento.

Os pacientes com DRC avançada, embora apresentem dificuldade para reconhecer e verbalizar suas emoções de maneira clara, expressam sua condição por meio de sentimentos de estresse, ansiedade, preocupações e medos, além de sintomas como dificuldade para dormir, cansaço e falta de energia. Os dados deste estudo corroboram a literatura existente. Uma pesquisa identificou sintomas depressivos em 87% dos pacientes, com 47% apresentando níveis graves ou extremamente graves da doença e 66,7% em estado grave ou extremamente grave. Em relação ao estresse, oito pacientes (53%) não apresentaram sintomas, enquanto 27% estavam em estágio grave, e nenhum paciente teve seu estresse classificado como extremamente grave^30^.

Um estudo sobre a qualidade de vida de pacientes com DRC avançada demonstrou que um plano terapêutico focado nos sintomas melhora significativamente a dor e a depressão. Além disso, a depressão e a fadiga afetam o autogerenciamento, reduzindo a motivação para a prática de atividades físicas, a adesão às recomendações dietéticas e a capacidade de lidar com o humor deprimido^31^.

Outro sintoma identificado neste estudo, após a aplicação do IPOS-Renal, foi a presença de dor em 35,9% dos entrevistados, sendo classificada como leve em 4,6%, moderada em 12,4%, forte em 24,8% e insuportável em 0,7%. Cicely Saunders, precursora dos cuidados paliativos, cunhou o termo “dor total” ao reconhecer, por meio das narrativas dos pacientes, que a dor vai além do aspecto físico, abrangendo dimensões emocionais, sociais e espirituais^32^. Desde então, os paliativistas consideram esses fatores na avaliação da dor, difundindo o conceito de dor total e sensibilizando os profissionais de saúde sobre a necessidade de compreendê-la como algo que vai além de um relato subjetivo, bem como de adotar medidas efetivas de compaixão e defesa dos direitos dos pacientes, garantindo um tratamento adequado, especialmente em ambientes de saúde^33,34^.

Os sintomas não controlados impactam negativamente as atividades diárias dos pacientes. Um estudo com 90 pacientes em diálise identificou a presença de dor musculoesquelética de intensidade moderada em 35,6% dos entrevistados^35^. Além disso, evidências indicam que a dor compromete a qualidade do sono^21,36,37^. Esses dados estão em consonância com os achados do presente estudo e com a literatura existente, uma vez que, entre os sintomas adicionais do IPOS-Renal, os pacientes relataram queixas de dor em locais específicos, como nos membros inferiores, além de dores generalizadas no corpo e nas articulações.

Para auxiliar no manejo da dor crônica, equipes especializadas em cuidados paliativos podem contribuir com uma abordagem integral e auxiliar no controle de sintomas persistentes^8^. No contexto da diálise, o controle da dor requer cautela, especialmente na escolha dos analgésicos, devido às particularidades farmacocinéticas desses pacientes^38^.

Em um estudo realizado com a aplicação do IPOS-Renal, foram identificados sintomas menos proeminentes, como náuseas e falta de ar. Achado semelhante foi observado no presente estudo, em que as queixas menos frequentes foram faltar de ar (7%), diarreia (14%) e vômitos (14%)^25^.

Contudo, a escala IPOS-Renal validada na língua tcheca^19^ apresentou a prevalência de alguns sintomas distintos dos observados nesta pesquisa. Os mais relatados incluíram boca seca ou dolorida, náuseas, falta de apetite e pernas inquietas. No entanto, houve similaridade nas queixas de dificuldade para dormir e fraqueza. Essas evidências reforçam a eficácia do IPOS-Renal na identificação das queixas de pacientes com DRC avançada.

Para avaliar os sintomas dos pacientes neste estudo e testar a validade convergente, além do IPOS-Renal, aplicou-se o instrumento ESAS-r. Os resultados indicaram a presença de sintomas de ansiedade, dor, alterações no bem-estar, redução do apetite, depressão, sonolência e cansaço. Destaca-se que os sintomas de dor, sonolência e cansaço também foram prevalentes na aplicação do IPOS-Renal, reforçando as evidências de sua validade convergente.

Contudo, apesar dos instrumentos disponíveis, ainda existem desafios na identificação das necessidades dos pacientes renais. Constatou-se, nesta investigação, que apenas um paciente mencionou disfunção sexual. Entretanto, uma investigação realizada no Brasil identificou a prevalência de disfunção erétil em 66,30% dos indivíduos em uma amostra de 98 pacientes^39^.

Em relação às mulheres em diálise, um estudo com 22 participantes revelou que, apesar de todas estarem em relações estáveis, apenas 7 (18%) mantinham vida sexual ativa, e nenhuma paciente com idade superior a 60 anos relatou atividade sexual^40^. Portanto, é fundamental fortalecer a relação entre os pacientes e a equipe de saúde, proporcionando um ambiente seguro para que possam expressar suas necessidades.

Os pacientes em hemodiálise precisam deslocar-se até os centros de tratamento três vezes por semana. Esse contato frequente com os profissionais favorece o estabelecimento de um vínculo com a equipe multiprofissional, o que pode facilitar a abordagem de queixas mais íntimas.

No entanto, neste estudo, as pesquisadoras não possuíam vínculo prévio com os entrevistados, sendo esse o primeiro contato, o que pode ter influenciado a ausência significativa de relatos sobre sexualidade, constituindo-se como uma limitação da pesquisa.

Diante do exposto, o apoio de uma equipe multiprofissional de cuidados paliativos é essencial para avaliar e atender às necessidades dos pacientes renais, especialmente à medida que a DRC avança, auxiliando-os na autogestão da doença e do tratamento.

Nesse contexto, a IPOS-Renal, aliada ao suporte de uma equipe especializada em cuidados paliativos e à colaboração da equipe multiprofissional da diálise, representa uma ferramenta valiosa para identificar e implementar um plano terapêutico capaz de minimizar o sofrimento dos pacientes.

## Conclusão

A IPOS-Renal demonstrou ser um instrumento válido, apresentando boa validade de conteúdo, confiabilidade e sensibilidade para detectar mudanças ao longo do tempo. Portanto, sua aplicação no Brasil mostra-se viável para a avaliação dos principais sintomas de pacientes com DRC avançada em cuidados paliativos. Seu uso não apenas possibilita uma avaliação mais precisa das necessidades dos doentes, mas também fortalece a comunicação entre a equipe de saúde e os pacientes, promovendo um cuidado mais centrado na pessoa e alinhado aos seus valores e desejos.

## Data Availability

Os dados que apoiam os achados deste estudo estão disponíveis mediante solicitação razoável à autora correspondente.

## Material Suplementar

O seguinte material online está disponível para o presente artigo:

Questionário do paciente – IPOS Renal.
